# Distinguishing chronic low back pain in young adults with mild to moderate pain and disability using trunk compliance

**DOI:** 10.1038/s41598-021-87138-6

**Published:** 2021-04-07

**Authors:** Alexander Stamenkovic, Brian C. Clark, Peter E. Pidcoe, Susanne M. van der Veen, Christopher R. France, David W. Russ, Patricia A. Kinser, James S. Thomas

**Affiliations:** 1 Department of Physical Therapy, College of Health Professions, Virginia Commonwealth University, 900 East Leigh St, 4th Floor, Richmond, VA 23298 USA; 2 Ohio Musculoskeletal and Neurological Institute (OMNI), Ohio University, Athens, USA; 3 Physical and Rehabilitation Medicine, Virginia Commonwealth University, Richmond, USA; 4 School of Physical Therapy & Rehabilitation Sciences, University of South Florida, Tampa, USA; 5 School of Nursing, Virginia Commonwealth University, Richmond, USA; 6 Department of Biomedical Sciences, Ohio University, Athens, USA; 7 Department of Psychology, Ohio University, Athens, USA

**Keywords:** Biomarkers, Musculoskeletal system, Chronic pain, Orthopaedics, Disability

## Abstract

Chronic low back pain (cLBP) rates among younger individuals are rising. Although pain and disability are often less severe, underlying changes in trunk behavior may be responsible for recurrence. We examine the biomarker capacity of a simple Trunk Compliance Index (TCI) to distinguish individuals with and without cLBP. A random subset (n = 49) of the RELIEF RCT were matched to healthy controls for sex, age, height and weight. We measured TCI (as displacement/ weight-normalized perturbation force) using anthropometrically-matched, suddenly-applied pulling perturbations to the trunk segment, randomized across three planes of motion (antero-posterior, medio-lateral, and rotational). Mean differences between cLBP, sex and perturbation direction were assessed with repeated-measures analysis of variance. Discriminatory accuracy of TCI was assessed using Receiver Operator Characteristic (ROC) analysis. Baseline characteristics between groups were equivalent (*x̅* [range]): sex (57% female / group), age (23.0 [18–45], 22.8 [18–45]), height, cm (173.0 [156.5–205], 171.3 [121.2–197], weight, kg (71.8 [44.5–116.6], 71.7 [46.8–117.5]) with cLBP associated with significantly lower TCI for 5 of 6 directions (range mean difference, − 5.35: − 1.49, range 95% CI [− 6.46: − 2.18 to − 4.35: − 0.30]. Classification via ROC showed that composite TCI had high discriminatory potential (area under curve [95% CI], 0.90 [0.84–0.96]), driven by TCI from antero-posterior perturbations (area under curve [95% CI], 0.99 [0.97–1.00]). Consistent reductions in TCI suggests global changes in trunk mechanics that may go undetected in classic clinical examination. Evaluation of TCI in younger adults with mild pain and disability may serve as a biomarker for chronicity, leading to improved preventative measures in cLBP.

Trial Registration and Funding RELIEF is registered with clinicaltrials.gov (NCT01854892) and funded by the NIH National Center for Complementary & Integrative Health (R01AT006978).

## Introduction

Low back pain, a leading cause of physician-sought care in the United States^1,2^, has been the largest global contributor to years lived with disability over the last three decades^3^. As the prevalence of low back pain rises, most notable are the increase in rates amongst younger adults^4,5^. A North Carolina population-based study^4^ showed a 200% rise in prevalence in adults 21–34 years old between 1992 and 2006. In the same sample, the increase among younger females was double that in males (320%); the greatest sex-based discrepancy across all ages^4^. Despite this, younger adults often present with mild to moderate pain and disability that may not justify dedicating interventional resources^6,7^. Considering the predictive nature of pain history on recurrence^8^, and lack of robust prediction rules for chronicity^9^, younger adults present an at-risk cohort that current diagnostic techniques address poorly^5,10^, and effective treatment strategies are hindered.

Clinician decisions relating to back pain are often informed by an interpretation of (1) patient response to specific movements (e.g., trunk flexion, extension), and (2) spinal mobility changes assessed through palpation^11–14^. Identifying a robust biomarker for chronic back pain using such assessments as a conceptual foundation may assist in (1) providing a target for intervention strategies (particularly if effective in a younger cohort), and (2) reducing the risk of increased disability in individuals as they approach middle age with persistent pain^4^.

Aligning with World Health Organization recommendations to establish biomarkers, and identify high risk groups for chronicity^15^, this complementary study to the RELIEF clinical trial^16^ investigated the ability of a Trunk Compliance Index, calculated using multi-planar trunk-based perturbations, to discriminate between young adults with mild to moderate pain and disability (drawn from the larger randomized controlled trial^16^, RCT) from matched controls. The Trunk Compliance Index was conceptually driven by clinician-based decisions on spinal mobility.

While compliance is classically defined as the inverse of stiffness and derived using more complex second-order linear modelling of the trunk, we explore the potential of a simple and clinically-translatable measure to spinal mobility by operationally defining the Trunk Compliance Index as the magnitude of displacement (from perturbation along the three cardinal planes of motion) divided by the normalized force. Given the inverse relationship of compliance to stiffness, evidence of increased spinal stiffness in males compared to females^17,18^, and increased stiffness in individuals with sub-acute^19^, recurrent^20^, and chronic low back pain^21,22^, we hypothesize lower levels of Trunk Compliance Indexes among (1) individuals with low back pain versus matched controls, and (2) males versus females.

## Methods

### Study design and cohort

This cross-sectional study used baseline data from a subset of individuals with low back pain collected during the Researching the Effectiveness of Lumbar Interventions for Enhancing Function (RELIEF) Study (clinicaltrials.gov: NCT01854892)^16^. RELIEF was an investigator-blinded, placebo-controlled RCT with 162 enrolled chronic back pain participants undergoing spinal manipulation and mobilization therapies over 6 treatments sessions during a 3-week period. Recruitment for RELIEF began June 1, 2013 and the primary completion date was August 31, 2017. The Ohio University Institutional Review Board approved this study with written informed consent obtained from all participants. This study followed the Strengthening the Reporting of Observational Studies in Epidemiology (STROBE) reporting guidelines.

Eligibility, inclusion and exclusion criteria for RELIEF have been described in detail previously^16^. Briefly, participants were required to satisfy minimum criteria for average pain > 2/10 on a numeric pain rating scale (with higher scores indicating greater pain) and disability (> 4/24 in the Roland Morris Disability Questionnaire, with higher scores indicating greater disability). During physical examinations, participants needed to meet 3 of 4 clinical characteristics associated with positive outcomes to spinal manipulation therapy (i.e., the primary intervention of RELIEF^16^).

A healthy control cohort, recruited from the local population through similar methods, was matched for sex, age, height and weight. Data collection for control participants occurred between October 10, 2016 and April 26, 2017. Data analysis for the current investigation occurred between March 1, 2020 and September 1, 2020.

### Data collection and procedures

Kinematic data were acquired at 100 Hz using a 10-camera motion capture system (Bonita, Vicon, Oxford, UK). Reflective marker clusters were affixed to axial (i.e., head, thorax, lumbar, pelvis) and appendicular body segments (upper arms, forearms, hands, thighs, shanks and feet).

**Figure 1:**
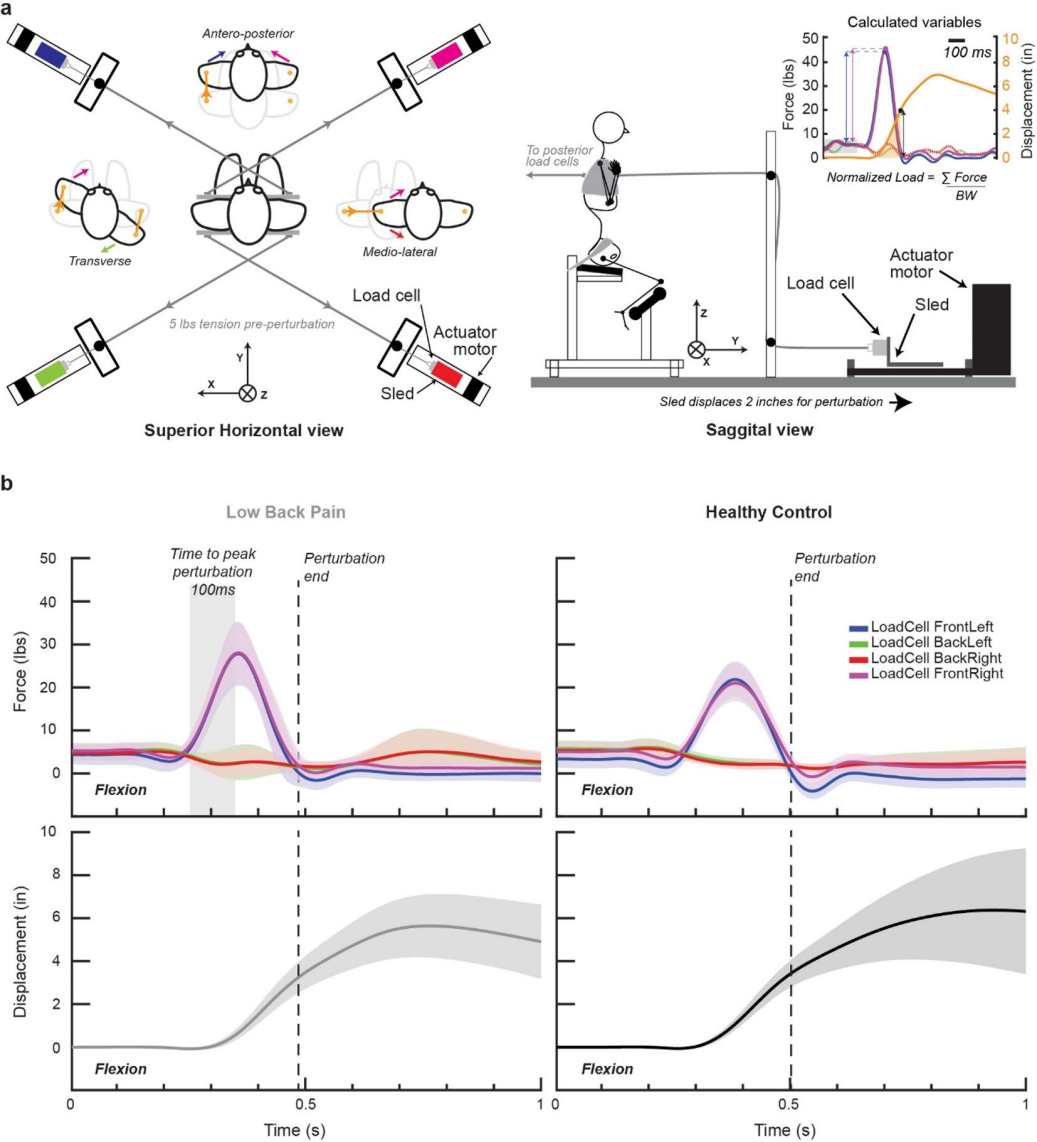
Experimental schematic of perturbation device and average time series data for calculation of Trunk Compliance Index. (**a**) Schematic of perturbation puller system with examples of motor combinations to produce directional perturbations and example calculation of measures, displacement and perturbation load. BW, Body weight. (**b**) Average time series profile of perturbation load (top panels) and displacement (bottom panels) for an individual with chronic low back pain (left panels) and matched control (right panel). Perturbations reached peak loads within 100 ms and displacement was calculated at the zero crossing following peak perturbation.

Suddenly-applied perturbations to the trunk were driven by four actuator motors (Parker, Oakboro, NC, USA) attached via light weight double braided polyester lines to a trunk harness (aligned with T3 vertebra) configured to allow 6 distinct directions of pull over the major axes of motion (i.e., flexion, extension, left- and right-side lateral flexion, and, clockwise and anti-clockwise rotation, Fig. 1a). Participants were centered and seated with the pelvis fixed and in neutral lumbar spine position within the perturbation system. Cables were pre-tensioned across actuator motors to a force-controlled 5 lbs (2.25 kg) prior to data acquisition and controlled using custom designed software in Labview (ver. 2012, National Instruments, Austin, USA). Force feedback was used to apply a sufficient impulse force to produce a 2 inch (5.08 cm) linear displacement within 100 ms. For each directional perturbation, one set of cable pairs pulled while the opposing pair released (see Fig. 1a). Following the perturbation, all lines were unladen. In total, participants were exposed to 18 perturbation trials, delivered at randomized time intervals following the beginning of data acquisition (range: 2–30 s) and randomized across direction (n = 3 trials/direction). Data was collected over eight seconds and synchronized through MotionMonitor (ver. xGen 3.42e, Innovative Sports, Chicago, IL). Offline post-processing of data was performed using customized scripts in MATLAB (ver. R2018b, The Mathworks, Natick, MA, USA). Kinematic and analog load cell data were low-pass filtered using a 41-point fourth-order Savitzky-Golay filter.

### Index of trunk compliance

As stiffness is defined as the resistance to deformation by an applied force (i.e., force/deformation)^23^, and compliance is the inverse of stiffness, we calculated a Trunk Compliance Index by dividing the magnitude of displacement due to perturbation, by the force of the applied perturbation load normalized to participant body weight.

Specifically, displacement was defined as the change in position of the left gleno-humeral joint center from baseline (a 200 ms period prior to perturbation application) to the zero crossing of perturbation force following peak perturbation force. Displacement examples are represented schematically in orange and as time series positional data in Fig. 1a, and as average time-series data for our two cohorts in Fig. 1b. Normalized perturbation load was calculated by summing the peak forces of the actuator motors responsible for the impulse load and dividing total force by participant body weight.

### Statistical analysis

Equivalences for age, height and weight between the low back pain and healthy control groups were assessed using Two One-Sided Test (TOST) procedures in the TOSTER package (ver. 0.4.6)^24^ in Excel (ver. 2016, Microsoft, Seattle, USA). Characteristics were deemed similar using the equivalence bounds of ± 1 year (age),  ± 4 in (height:  ~ 10 cm), and ± 5 lbs (weight:  ~ 2.25 kg).

Trunk Compliance Index was assessed with a four-way mixed methods repeated measures ANOVA (Within-subjects: Direction, Trial; Between-subjects: Low back pain, Sex) to determine whether pain- and sex-related changes were present in response to sudden multi-directional perturbations. Significance in the planned three-way interaction of interest (i.e., Direction x Sex x Pain Condition) was assessed with 4 separate univariate repeated measures ANOVAs split across factor levels. To reduce the family-wise error rate, Bonferroni-Holm adjustments were applied to main ANOVA results before determining significance (such that *P* = 0.05 / 4). Observed violations to sphericity were adjusted using Greenhouse–Geisser corrections. Significant interactions from univariate analyses were assessed using post-hoc procedures with Bonferroni adjustment for multiple pairwise comparisons. All ANOVA procedures were performed using SPSS (ver. 26, IBM, Armonk, NY). Two-sided significance was set at adjusted *P* < 0.05.

The potential of the Trunk Compliance Index in classifying individuals with and without chronic back pain was summarized using receiver operating characteristic (ROC) curves. ROC curves can estimate the discriminatory capability of biomarkers, and have been used previously in the context of low back pain^25,26^. with higher area under the curve (AUC) values representing greater biomarker accuracy in subject classification. Cut-off values were defined using the maximum Youden index (*J*_*max*_), the farthest point of the ROC curve from the reference line (set at 0.5, and indicating a discriminatory capacity no greater than chance).

## Results

### Participant characteristics

Of 162 participants enrolled in RELIEF^16^, 49 participants (57% female) with a mean (*SD*) age of 23.0 (5.4) years (range, 18–45) were randomly assigned at baseline to undergo trunk perturbations. Clinical characteristics between the RELIEF cohort and perturbation sub-group were within minimal clinically important differences.

The perturbation sub-group was also equivalent in age (mean difference: − 0.14 years), height (− 0.65 cm), and weight (− 0.05 kg) to the 49 matched control participants (57% female). Cohort characteristics and equivalence testing between the low back pain group and healthy control group are reported in Table 1.

**Table 1:** Baseline participant characteristics.

*Variable*	Low back pain				Healthy control				Difference of matched pairs					
	n = 49 (57% female)				n = 49 (57% female)						*Two one-sided tests for equivalence*			
	$\overline{x}$	s	min	max	$\overline{x}$	s	min	max	$\overline{x}$	s	Bounds (2-sided)	Observed Cohen's d	t_(48)_	*P*
Age (years)	23.0	5.4	18.0	45.0	22.8	5.7	18.0	45.0	− 0.1	2.6	1	0	− 2.70	*0.005*
Height (cm)	173.0	10.0	156.5	205.0	171.3	15.5	121.2	197.0	− 0.7	3.9	10	0	2.44	*0.009*
Weight (kg)	71.8	16.5	44.5	116.6	71.7	16.4	46.8	117.5	− 0.1	11.5	2.25	0	3.00	*0.002*
Body Mass Index (kg/m^2^)	24.7	5.4	18.4	49.0	24.0	4.0	18.5	34.6						
LBP duration (months)	64.41	49.34	4	275	–	–	–	–						

### Trunk Compliance Index

Load cell data from 6/1764 trials (n = 3, RELIEF perturbation sub-group; 2 female, 1 male) were missing due to corruption in analog signals. A sensitivity analysis was conducted using multiple imputation for missing indexes using SPSS. Comparison of results from the original analysis and imputed datasets (n = 5) showed no differences in significance of the planned interaction therefore, summary statistics and results are reported for the original dataset and analysis plan (see Table 2).

**Table 2:** Comparison of Trunk Compliance Index between pain condition and sex.

	Female (n = 26, 57%)						Male (n = 20, 43%)						Dir.*Pain (Female)			Dir.*Pain (Male)		
Variable	n	$\overline{x}$	s	95% CI	min	max	n	$\overline{x}$	s	95% CI	min	Max	$\overline{x}$	SE	95% CI	$\overline{x}$	SE	95% CI
**Low Back Pain (n = 46)**													**Group difference**					
**Trunk Compliance Index**																		
Flexion	83	8.47	1.69	[8.10, 8.84]	4.36	12.32	62	9.14	2.03	[8.63, 9.66]	5.14	14.26	− 5.25	0.45	[− 6.14, − 4.35]	− 5.35	0.52	[− 6.38, − 4.32]
Extension	84	7.96	1.84	[7.56, 8.36]	3.01	12.09	61	9.25	2.40	[8.64, 9.86]	3.61	15.85	− 5.15	0.48	[− 6.11, − 4.18]	− 4.73	0.56	[− 5.83, − 3.62]
Lat. flexion (Right)	83	7.41	1.60	[7.06, 7.76]	4.92	15.98	63	8.10	2.06	[7.58, 8.62]	4.43	14.39	0.36	0.40	[− 0.43, 1.14]	− 0.24	0.45	[− 1.15, 0.66]
Lat. flexion (Left)	84	5.66	1.37	[5.36, 5.95]	2.86	9.35	63	6.67	1.73	[6.23, 7.10]	2.16	11.25	− 1.49	0.35	[− 2.18, − 0.80]	− 1.77	0.40	[− 2.56, − 0.98]
Rotation (Anti-C)	84	12.71	3.57	[11.93, 13.48]	3.21	21.46	63	11.45	2.38	[10.85, 12.05]	6.43	16.24	− 1.69	0.70	[− 3.07, − 0.30]	− 4.87	0.80	[− 6.46, − 3.29]
Rotation (C)	83	12.51	3.28	[11.80, 13.23]	5.84	20.74	63	13.16	3.29	[12.33, 13.99]	7.38	25.77	− 1.51	0.77	[− 3.04, 0.11]	− 2.88	0.88	[− 4.63, − 1.13]

	Female (n = 28, 57%)						Male (n = 21, 43%)						Dir.*Sex (Low back pain)			Dir.*Sex (Healthy Control)		
	n	$\overline{x}$	s	95% CI	min	max	n	$\overline{x}$	s	95% CI	min	max	$\overline{x}$	SE	95% CI	$\overline{x}$	SE	95% CI
	**Healthy Control (n = 49)**												**Group difference**					
Flexion	84	13.59	1.92	[13.17, 14.00]	10.18	20.57	63	14.44	1.84	[13.98, 14.91]	10.75	21.71	− 0.75	0.50	[− 1.76, 0.26]	− 0.86	0.47	[− 1.80, 0.09]
Extension	84	13.12	1.88	[12.72, 13.53]	8.66	19.06	63	13.94	1.73	[13.50, 14.38]	10.17	17.67	− 1.24	0.59	[− 2.43, − 0.05]	− 0.82	0.45	[− 1.72, 0.09]
Lat. flexion (Right)	84	7.00	1.10	[6.76, 7.24]	4.24	9.90	63	8.30	1.20	[7.99, 8.60]	6.00	10.98	− 0.69	0.54	[− 1.77, 0.39]	− 1.29	0.30	[− 1.89, − 0.69]
Lat. flexion (Left)	84	7.05	1.08	[6.82, 7.29]	4.01	10.34	63	8.40	1.22	[8.10, 8.71]	5.59	11.54	− 1.07	0.44	[− 1.95, − 0.18]	− 1.35	0.30	[− 1.96, − 0.74]
Rotation (Anti-C)	84	14.13	2.71	[13.54, 14.72]	8.74	22.60	63	16.11	3.17	[15.31, 16.91]	9.74	23.67	1.20	0.81	[− 0.43, 2.84]	− 1.98	0.69	[− 3.38, − 0.59]
Rotation (C)	84	14.03	2.57	[13.47, 14.59]	9.21	19.66	63	15.99	2.91	[15.25, 16.72]	10.35	20.93	− 0.59	0.91	[− 2.43, 1.24]	− 1.96	0.74	[− 3.45, − 0.47]

**Figure 2:**
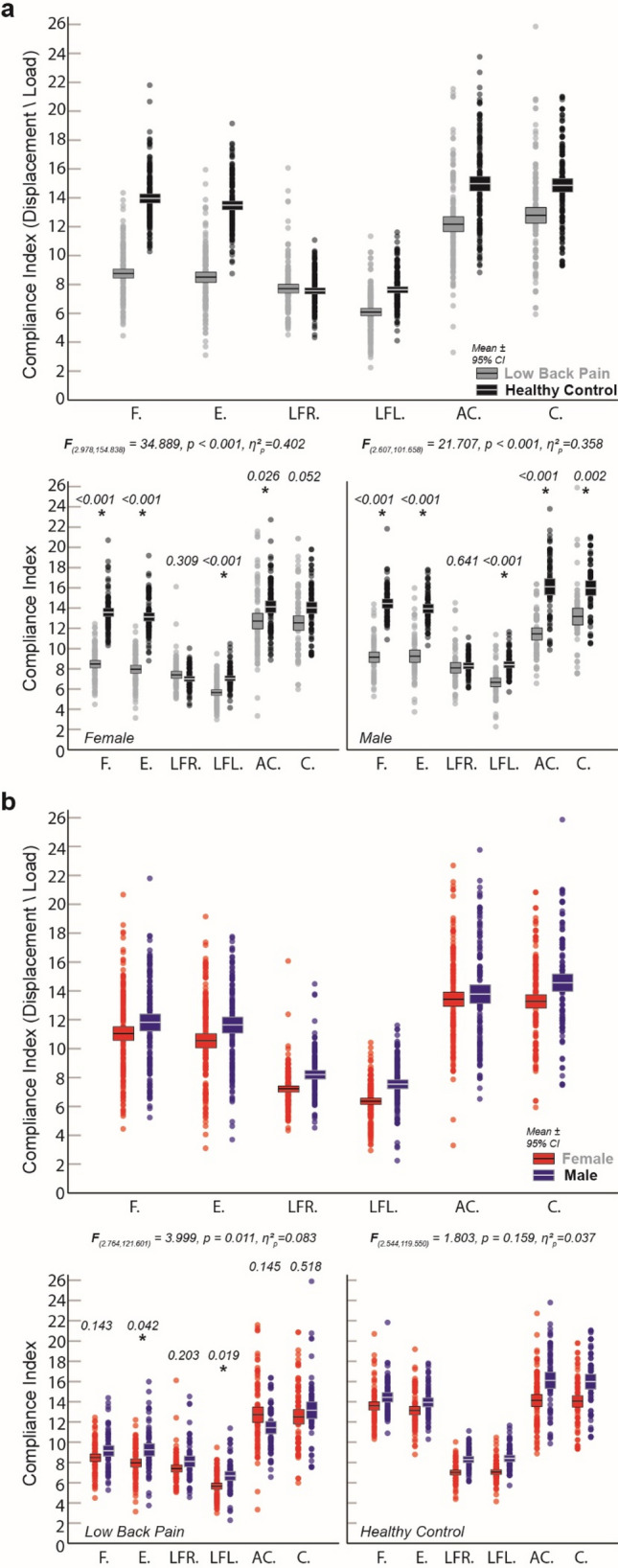
Mean differences in Trunk Compliance Index across 6 directions of trunk position perturbation. (**a**) Comparisons were split by chronic low back pain (a, grey) and matched healthy controls (a, black). (**b**) Color comparisons were split by biological sex (females, red; males, blue). *P < 0.05 Bonferroni-adjusted; All individual values reported with bars representing mean + 95% confidence interval. Abbreviations: F, Flexion; E, Extension; LFR, Lateral Flexion (Right); LFL, Lateral Flexion (Left); AC, Anti-clockwise (rightward) rotation; C, Clockwise (leftward) rotation.

A significant three-way interaction (Direction of perturbation × Sex × Pain Condition: [F_(2.924, 266.053)_ = 4.937, *P* = 0.003, η^2^_p_ = 0.051]) was driven by lower Trunk Compliance Indexes across 5 of 6 perturbation directions for the chronic low back pain group (Fig. 2a, Direction x Pain Condition: Female [F_(2.978, 154.838)_ = 34.889, *P* < 0.001, η^2^_p_ = 0.402]; Male: F_(2.607, 101.658)_ = 21.707, *P* < 0.001, η^2^_p_ = 0.358]) and across Sex (Fig. 2b, Direction × Sex: LBP [F_(2.764, 121.601)_ = 3.999, *P* = 0.011, η^2^_p_ = 0.083]; Healthy Control: F_(2.544, 119.550)_ = 1.803, *P* = 0.159, η^2^_p_ = 0.037]).

Lower Trunk Compliance Indexes (in/(%weight)) were present in both females and males with chronic back pain with mean group differences [95% CI] for flexion (females: − 5.25, [− 6.14, − 4.35], *P* < 0.001; males: − 5.35, [− 6.38, − 4.32], *P* < 0.001), extension (females: − 5.15, [− 6.11, − 4.18], *P* < 0.001; males: − 4.73, [− 5.83, − 3.62], *P* < 0.001), leftward lateral flexion (females: − 1.49, [− 2.18, − 0.80], *P* < 0.001; males: − 1.77, [− 2.56, − 0.98], *P* < 0.001), and anti-clockwise rotation (females: − 1.69, [− 3.07, − 0.30], *P* = 0.018; males: − 4.87, [− 6.46, − 3.29], *P* < 0.001) perturbations. Males with low back pain presented lower Trunk Compliance Indexes for clockwise rotation perturbations (males: − 2.88, [− 4.63, − 1.13], *P* = 0.002) that were not evident in females (females: − 1.51, [− 3.04, 0.11], *P* = 0.052). Sex or pain factors were not associated with significant changes to Trunk Compliance Indexes for rightward lateral flexion perturbations (females: 0.36, [− 0.43, 1.14], *P* = 0.370; males: − 0.24, [− 1.15, 0.66], *P* = 0.592).

Sex-based differences were less robust, with females in the chronic back pain group showing significantly lower indexes than male counterparts for extension (LBP: − 1.24, [− 2.43, − 0.05], *P* = 0.042) and leftward lateral flexion perturbations (LBP: − 1.07, [− 1.95, − 0.18], *P* = 0.019).

### Classifying chronic back pain: receiver operating characteristic (ROC) analysis

**Figure 3:**
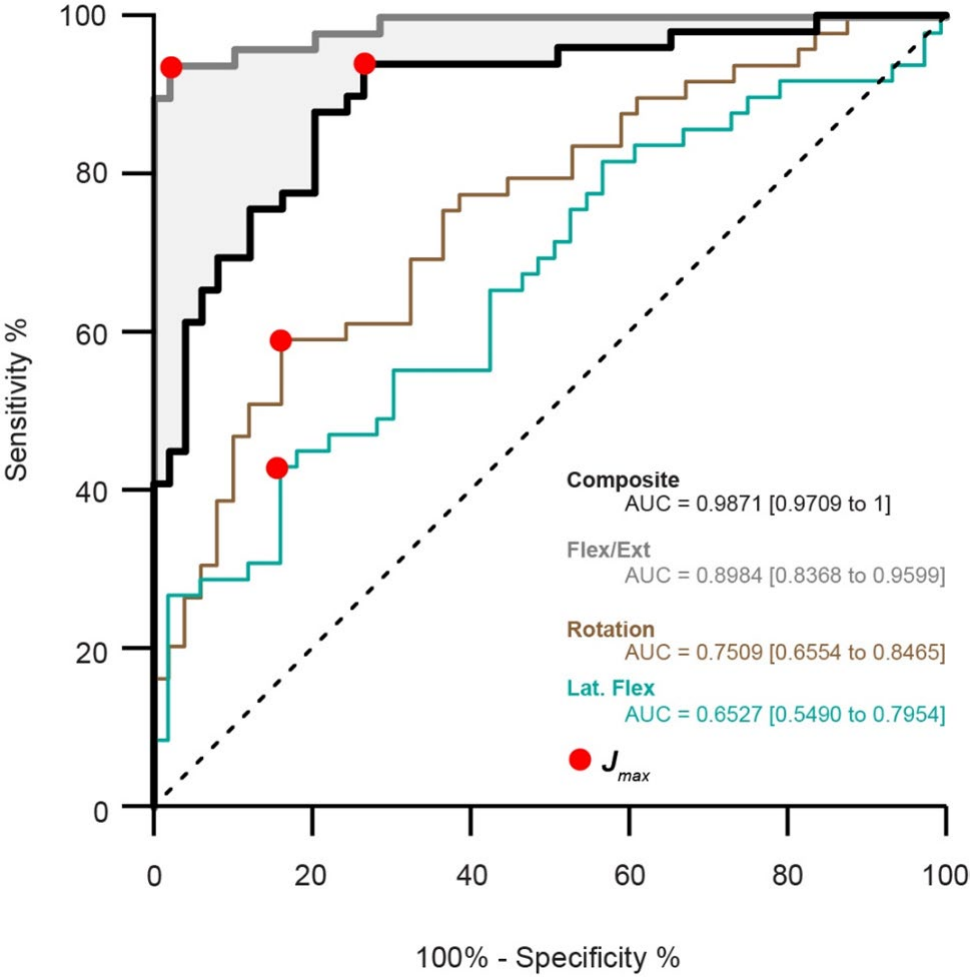
Receiver Operator Characteristic (ROC) curves for Trunk Compliance Index. Summary curves were produced for a Composite Trunk Compliance Index score (averaged across all perturbation directions, black), and individually for perturbations across each plane of motion (Flexion/Extension-grey; lateral flexion-blue; Rotation-brown). Area under the curve (AUC), and Youden’s index (J_*max*_) indicating optimal cut-points are shown for measures with good to excellent discriminatory capacity.

Summary ROC analyses across each plane of motion, and averaged across all conditions to create a composite score, are presented in Fig. 3. Composite scores showed excellent^27^ discriminatory capacity (AUC [95% CI], 0.898 [0.837–0.960]) for chronic back pain with high sensitivity (93.88 [95% CI, 83.48–97.90]) and specificity (73.47 [59.74–83.79]) reflecting a ~ 3.5 × greater likelihood of exhibiting Trunk Compliance Indexes below 11.42 (*J*_*max*_, 11.42; positive likelihood ratio, 3.538).

When separated by plane of motion, antero-posterior perturbations provided outstanding^27^ discriminatory capabilities (AUC, 0.9871 [0.9709–1], Fig. 3, grey) with greater sensitivity (93.88 [83.48–97.90]) and specificity (97.96 [89.31–99.90]) measures that were 46 × more likely to exhibit Trunk Compliance Indexes below 11 (*J*_*max*_ = 10.97; positive likelihood ratio = 46.0). Other planar perturbations directions were less effective discriminators between groups (Medio-lateral: AUC, 0.6527 [0.5490–0.7954]; Sensitivity, 42.86 [30.02–56.73]; Specificity, 83.67 [70.96–91.49]; Positive likelihood ratio, 2.625); Rotation: AUC, 0.7509 [0.6554–0.8465]; Sensitivity, 59.18 [45.25–71.78]; Specificity 83.67 [70.96–91.49]; Positive likelihood ratio, 3.625).

## Discussion

When applied across three cardinal planes of motion, rapid position-based perturbations of the trunk revealed decreased Trunk Compliance Indexes in relatively young individuals with chronic back pain compared to controls matched for sex, age, height and weight (Fig. 2a). Reduced Trunk Compliance Indexes persisted across 5 of 6 perturbation conditions, echoing studies showing increased effective stiffness in back pain cohorts perturbed in a single plane (e.g., sub-acute^19^, recurrent^20^). Although females exhibited decreased Trunk Compliance Indexes (Fig. 2b), sex-based differences were not robust across perturbation directions. Females with chronic back pain showed decreased Trunk Compliance Indexes in extension and left lateral flexion perturbations. This was in contrast to our original hypothesis, as previous work has identified higher intrinsic compliance in females compared to their male counterparts^17,18^. However, results of sex-based differences in effective trunk compliance remain mixed when comparing methodological approaches (e.g., force vs. position-constant perturbation^28,29^) and a continued focus of sex-based differences remains warranted.

Younger adults with chronic back pain are an underserved cohort because they often have mild symptom severity and disability, and typically maintain functional capacity. These factors inform clinical decisions and divert interventional resources towards older populations where pain severity leads to significant disability^6,7^. However, mechanical trunk changes apparent in the current study despite minimal impact on pain, disability and function^16^, highlight the need to re-evaluate clinical decision processes. The allocation of resources based on disability alone ignores known benefits of early intervention on pain trajectories in back pain populations^30^. Further, our results suggest a global change in trunk behaviour that may not emerge from classic clinical and subjective evaluations of trunk motion or spinal palpation, but which could leverage such assessments when standardized and quantified appropriately.

Prior work points to the potential generalisability of our findings from younger adults with mild pain and disability^16^ to the greater chronic back pain population. Regarding presentation and treatment efficacy, changes in baseline characteristics and prognostic outcomes over 12 months show age-related differences between younger and older cohorts that fall within the bounds of minimal clinical relevance^31^. Younger and older adults also show similar responses to conservative treatment options^32^. Further, although age-related degeneration of passive structures have shown decreased stiffness in cadaveric-based^33^ and structural imaging examinations^34,35^, recent functional-based findings are mixed, showing no evidence^18^, or increased levels of intrinsic stiffness as a function of aging^36^. While in healthy individuals^36^, age-related increases in stiffness are postulated to stem from greater trunk co-activation mediated by psychosocial factors (i.e., stress^37^). Therefore, the biopsychosocial nature of chronic back pain, coupled with evidence of a weak (but present) association of kinesiophobia to increases in intrinsic trunk stiffness to perturbation^38^, would suggest older individuals with chronic back pain would have similar, if not greater changes in trunk compliance exacerbated by these additional confounds.

Biomarkers are key to the pursuit of stratifying disease progression and prognosis in chronic back pain. To date, primary areas of foci often require minimally-invasive procedures (e.g., blood-based markers) to examine genetic or pro-inflammatory cytokine associations with pain severity, with mixed diagnostic success^39,40^.

The current study provides a method to investigate measures with biomarker promise that (1) can be obtained non-invasively and (2) may functionally reflect underlying global alterations of active and passive trunk elements associated with chronic back pain. Other work examining discriminatory accuracy in a similar fashion is limited. Two examples includes the Foundation Pain Index (FPI), a proprietary algorithm based on an assay of 11 urinary based pain markers^26^, and the questionnaire-based Subgroup for Targeted Treatment Back Screening Tool (STarTBack), which classifies patients into low, medium or high risk sub-groups of persistent disabling back pain^41^. Comparing the discriminatory capacity of the Trunk Compliance Index in the current study (AUC = 0.89), the FPI (AUC, 0.749), and STarTBack (AUC, 0.71–0.84), show reduced discriminatory capacity. In fact, the STarTBack showed that simple physiological measures including trunk endurance and maximal voluntary contraction provided little discriminatory capacity^41^.

Trunk compliance (and inversely, stiffness) is non-linear and strictly defined from a mechanical perspective. Therefore, our measure of Trunk Compliance Index provides an approximation of a complex metric. Methodological decisions are also known to influence the calculation of stiffness and compliance^17,20^. In the current study, confounds were minimized by (1) using positional-based perturbations to mitigate trunk kinematic differences^17^, (2) reducing the effect of muscle preload prior to perturbation using pre-tensioning^20^, and (3) providing a perturbation time course (100 ms rise to peak) that reduces the potential for active voluntary contributions to influence our primary measure of trunk compliance.

Often in the examination of stiffness an attempt is made to dissociate elements that contribute to effective stiffness of the system (e.g., intrinsic passive, active reflexive and volitional contributions to stiffness). While previous work has calculated effective stiffness over extended time courses (~ 350 ms)^20^, our study provided 100 ms perturbation and examination of motion over a 200 ms period from perturbation initiation. These values lie well within the realm of examinations that limit active voluntary but not active reflexive contributions^42^. While studies have found mixed results to active reflex latencies across trunk perturbations, a meta-analysis^42^ reported increased latencies in erector spinae activity in low back pain individuals. Therefore, while reflex latencies were not analysed in the current study, their apparent delay in low back pain populations would suggest that they might provide a small representation in our measure of trunk compliance. While ROC curves showed good to excellent discriminatory capacity for our composite score and antero-posterior perturbations, it must also be acknowledged that this analysis was exploratory and ROC analyses are often conducted with greater sample size considerations. Future work should consider the inclusion of patient-specific metrics including site and location of pain to improve the discriminatory capability of lateral and rotational components of the Trunk Compliance Index (and observed changes for rotation-based perturbations), which may be attributable to asymmetries in presentation.

These findings provide the first step towards the development of a Trunk Compliance Index to distinguish individuals with chronic low back pain. By providing ‘proof of concept’ and face validity for this measure, further development can prioritize reliability testing, including for repeatability of outcomes across raters, and sensitivity of changes over time. Additionally, further validation in other domains (e.g., comprehensive testing of construct, content and criterion validity) should follow iterative design changes to the apparatus that package the current concepts of trunk compliance tested into a device with a clinically feasible footprint.

## Conclusion

Lower levels in Trunk Compliance Index were evident and preserved across perturbation movement directions in young individuals with mild to moderate chronic low back pain as compared to matched controls. This signifies a potential global marker of trunk behaviour that is present despite limited influences of pain and disability on function. Therefore, a simple metric of trunk compliance, conceptually driven by clinician-based decisions on spinal mobility, may be leveraged by future work as a method to assess outcomes of interventions that are biopsychosocial in nature.

## Disclaimer

The content is solely the responsibility of the authors and does not necessarily represent the official views of the NIH.
