# Publisher Correction: Distinguishing chronic low back pain in young adults with mild to moderate pain and disability using trunk compliance

**DOI:** 10.1038/s41598-021-95583-6

**Published:** 2021-08-03

**Authors:** Alexander Stamenkovic, Brian C. Clark, Peter E. Pidcoe, Susanne M. van der Veen, Christopher R. France, David W. Russ, Patricia A. Kinser, James S. Thomas

**Affiliations:** 1 Department of Physical Therapy, College of Health Professions, Virginia Commonwealth University, 900 East Leigh St, 4th Floor, Richmond, VA 23298 USA; 2 Ohio Musculoskeletal and Neurological Institute (OMNI), Ohio University, Athens, USA; 3 Physical and Rehabilitation Medicine, Virginia Commonwealth University, Richmond, USA; 4 School of Physical Therapy & Rehabilitation Sciences, University of South Florida, Tampa, USA; 5 School of Nursing, Virginia Commonwealth University, Richmond, USA; 6 Department of Biomedical Sciences, Ohio University, Athens, USA; 7 Department of Psychology, Ohio University, Athens, USA

Correction to: *Scientific Reports* 10.1038/s41598-021-87138-6, published online 07 April 2021

The original version of this Article contained an error in Table 2 where the row containing values for “Rotation (C)” was omitted for the Low Back Pain cohort.

The original Table 2 and accompanying legend appear below.

**Table 2:** Comparison of Trunk Compliance Index between pain condition and sex.

Variable	Low back pain (n = 46)												Group difference					
	Female (n = 26, 57%)						Male (n = 20, 43%)						Direction*Pain			Direction*Pain		
													Female			Male		
	n	$\overline{x}$	s	95%CI	min	max	n	$\overline{x}$	s	95%CI	min	max	$\overline{x}$	SE	95% CI	$\overline{x}$	SE	95% CI
**Trunk compliance index**																		
Flexion	83	8.47	1.69	[8.10, 8.84]	4.36	12.32	62	9.14	2.03	[8.63, 9.66]	5.14	14.26	− 5.25	0.45	[− 6.14, − 4.35]	− 5.35	0.52	[− 6.38, − 4.32]
Extension	84	7.96	1.84	[7.56, 8.36]	3.01	12.09	61	9.25	2.40	[8.64, 9.86]	3.61	15.85	− 5.15	0.48	[− 6.11, − 4.18]	− 4.73	0.56	[− 5.83, − 3.62]
Lat. flexion (Right)	83	7.41	1.60	[7.06, 7.76]	4.92	15.98	63	8.10	2.06	[7.58, 8.62]	4.43	14.39	0.36	0.40	[− 0.43, 1.14]	− 0.24	0.45	[− 1.15, 0.66]
Lat. flexion (Left)	84	5.66	1.37	[5.36, 5.95]	2.86	9.35	63	6.67	1.73	[6.23, 7.10]	2.16	11.25	− 1.49	0.35	[− 2.18, − 0.80]	− 1.77	0.40	[− 2.56, − 0.98]
Rotation (Anti-C)	84	12.71	3.57	[11.93, 13.48]	3.21	21.46	63	11.45	2.38	[10.85, 12.05]	6.43	16.24	− 1.69	0.70	[− 3.07, − 0.30]	− 4.87	0.80	[− 6.46, − 3.29]

	Healthy control (n = 49)												Direction*Sex			Direction*Sex		
	Female (n = 28, 57%)						Male (n = 21, 43%)											
													cLBP			Healthy Controls		
	n	$\overline{x}$	s	95%CI	min	max	n	$\overline{x}$	s	95%CI	min	max	$\overline{x}$	SE	95% CI	$\overline{x}$	SE	95% CI
Flexion	84	13.59	1.92	[13.17, 14.00]	10.18	20.57	63	14.44	1.84	[13.98, 14.91]	10.75	21.71	− 0.75	0.50	[− 1.76, 0.26]	− 0.86	0.47	[− 1.80, 0.09]
Extension	84	13.12	1.88	[12.72, 13.53]	8.66	19.06	63	13.94	1.73	[13.5, 14.38]	10.17	17.67	− 1.24	0.59	[− 2.43, − 0.05]	− 0.82	0.45	[− 1.72, 0.09]
Lat. flexion (Right)	84	7.00	1.10	[6.76, 7.24]	4.24	9.90	63	8.30	1.20	[7.99, 8.60]	6.00	10.98	− 0.69	0.54	[− 1.77, 0.39]	− 1.29	0.30	[− 1.89, − 0.69]
Lat. flexion (Left)	84	7.05	1.08	[6.82, 7.29]	4.01	10.34	63	8.40	1.22	[8.1, 8.71]	5.59	11.54	− 1.07	0.44	[− 1.95, − 0.18]	− 1.35	0.30	[− 1.96, − 0.74]
Rotation (Anti-C)	84	14.13	2.71	[13.54, 14.72]	8.74	22.60	63	16.11	3.17	[15.31, 16.91]	9.74	23.67	1.20	0.81	[− 0.43, 2.84]	− 1.98	0.69	[− 3.38, − 0.59]
Rotation (C)	84	14.03	2.57	[13.47, 14.59]	9.21	19.66	63	15.99	2.91	[15.25, 16.72]	10.35	20.93	− 0.59	0.91	[− 2.43, 1.24]	− 1.96	0.74	[− 3.45, − 0.47]

The original Article has been corrected.

